# Express penetration of hydrogen on Mg(10͞13) along the close-packed-planes

**DOI:** 10.1038/srep10776

**Published:** 2015-06-01

**Authors:** Liuzhang Ouyang, Jiajun Tang, Yujun Zhao, Hui Wang, Xiangdong Yao, Jiangwen Liu, Jin Zou, Min Zhu

**Affiliations:** 1 School of Materials Science and Engineering, Key Laboratory of Advanced Energy Storage Materials of Guangdong Province, South China University of Technology, Guangzhou, 510641, People’s Republic of China; 2 Department of Physics, South China University of Technology, Guangzhou 510640, People’s Republic of China; 3 School of Biomolecular and Physical Sciences, Griffith University, Nathan, QLD 4111, Australia; 4 China-Australia Joint Laboratory for Energy & Environmental Materials, South China University of Technology, Guangzhou, 510641, PR China; 5 Materials Engineering and Centre for Microscopy and Microanalysis, The University of Queensland, St. Lucia, QLD 4072, Australia

## Abstract

Metal atoms often locate in energetically favorite close-packed planes, leading to a relatively high penetration barrier for other atoms. Naturally, the penetration would be much easier through non-close-packed planes, i.e. high-index planes. Hydrogen penetration from surface to the bulk (or reversely) across the packed planes is the key step for hydrogen diffusion, thus influences significantly hydrogen sorption behaviors. In this paper, we report a successful synthesis of Mg films in preferential orientations with both close- and non-close-packed planes, i.e. (0001) and a mix of (0001) and (10
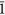
3), by controlling the magnetron sputtering conditions. Experimental investigations confirmed a remarkable decrease in the hydrogen absorption temperature in the Mg (10
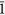
3), down to 392 K from 592 K of the Mg film (0001), determined by the pressure-composition-isothermal (PCI) measurement. The *ab initio* calculations reveal that non-close-packed Mg(10
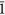
3) slab is advantageous for hydrogen sorption, attributing to the tilted close-packed-planes in the Mg(10
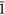
3) slab.

Mg-based materials are very attractive for hydrogen storage^1^,^2^,^3^,^4^,^5^ due to its natural abundance and high hydrogen storage density, i.e. nearly 7.6 wt.% or 0.11 kg H_2_L^−1^. However, their applications suffer from very high thermodynamics stability and kinetics barrier^1^. Tremendous efforts including catalysis, nanostructuring and alloying have been devoted to tune these two properties^1^,^4^. It has been reported that the effects related to interfaces or surfaces may substantially influence the thermodynamics and kinetics that determine the hydrogen storage performance^4^. For example, the hydrogen absorption temperature plummets to 373 K in a three-layered Pd/Mg/Pd film^5^. When replace the precious Pd with AB_3_ or AB_5_ hydrogen storage alloys in multilayer films, the hydrogen sorption temperature was also significantly reduced^6^. It is also reported that there is a significant increase (about 200 times) in equilibrium hydrogen pressure in Mg thin films capped with Pd^7^, and the elastic strain energy brought by the clamping effect of the Pd cap plays an important role^7^,^8^.

The mechanisms for improvement of hydrogen storage properties related to the interfacial effect have generally been attributed to the strain effect^6^, interfacial phases^7^, spilling over effect^4^ and interfacial energy change^1^. However, the role of the crystallographic feature of the interface or surface has not been elucidated yet. Close-packed planes are energetically propitious for metal atom settlement, such as (111) for fcc crystals, e.g.MgH_2_, and (0001) for hcp crystals, e.g. Mg. Generally, due to that atoms often locate in energetically favorite close-packed planes, a low-index metallic film will be fabricated by self-assembly of metal atoms under a normal condition to minimize the energy^9^,^10^ This low-index film with close-packed planes is consisting of high density of atoms and small vacancies^11^, which is energetically unfavorable for other atoms to penetrate. High temperature is necessary for hydrogenation/dehydrogenation in metals, e.g. Mg, due to the high penetration barrier. This is a critical drawback to hinder the utilization of metal hydrides in hydrogen storage applications.

It is well known that the crystallographic feature determines the atoms relocation at the interface or surface, which has significant effects on material behaviors, such as hydrogen sorption^12^,^13^ or interface polarization^14^,^15^. For instance, it is reported that the specific energy of hydrogen adsorption on the surface of palladium single crystals is depended on the surface orientations, e.g. 24.4 and 20.8 kcal/mole for Pd (110) and Pd (111) surfaces, respectively^16^. It is also observed that the energy varies with different surface orientations of Ni^17^. In particular for MgH_2_^18^, the theoretical calculations reveals that the energy barriers of H_2_ desorption are associated with the crystalline surfaces, e.g. MgH_2_(001) and MgH_2_(110). The energy barrier for hydrogen sorption in higher index of MgH_2_(110) is lower than that of MgH_2_(001) surface.

Accordingly, it is hypothesized that if can control the preferential orientation of Mg surfaces, e.g. non-close-packed versus close-packed surfaces, the hydrogen sorption performance in Mg could be modified through its crystallographic features. Here, we present a feasible approach to control the preferential orientation of Mg films, i.e. (0001) and a mix of (0001) and (10
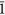
3). It is demonstrated that the high-index (10
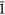
3) crystal surface can adsorb hydrogen at a temperature much lower than that of the low-index (0001) crystal surface, which is attributed to the non-close-packed planes in Mg(10
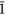
3) that allow H atoms to penetrate with low energy barrier, as revealed by theoretical calculations.

## Results and Discussion

**Figure 1:**
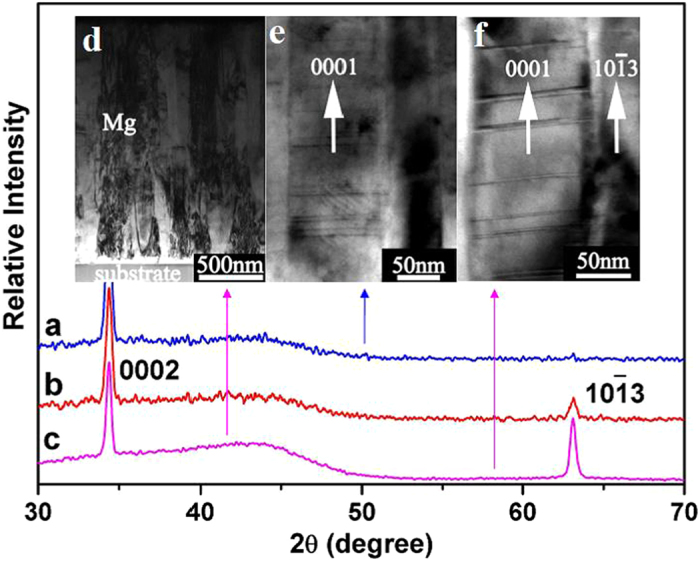
X-ray diffraction (XRD) patterns of Mg thin film deposited at different Ar gas pressure. Mg thin film deposited at (**a**) 1.4 × 10^−2^ Pa, (**b**) 5.6 × 10^−1^ Pa and (**c**) 1.4 × 10^−1^ Pa, respectively. (**d**)XTEM bright field images showing general morphology of the Mg thin film, (**e**) and (**f**) are higher magnification of film with Mg (0001) texture and (0001) + (10
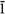
3) texture, respectively.

Figure 1(a–c) show the XRD patterns of the Mg thin films deposited onto the substrate using 250 W sputtering power under 1.4 × 10^−2^ Pa, 5.6 × 10^−1^ Pa, and 1.4 × 10^−1^ Pa Ar gas pressures, respectively. It is shown that only one peak is observed, corresponding to the (0001) reflection of the Mg thin film deposited under 1.4 × 10^−2^ Pa Ar gas pressure. The Mg thin film fabricated under such a condition is with a highly preferential (0001) orientation, which is a quite common case in the deposition of the monolayer and multilayer Mg films^19^. However, it is observed in Fig. 1 (b) and (c) that two peaks are presented, corresponding to the (0001) and (10
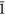
3) reflections, respectively, under the pressures of 5.6 × 10^−1^ Pa and 1.4 × 10^−1^ Pa. The intensity ratio of the (0002) to (10
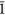
3) peak is dependent on the Ar gas pressure. This demonstrates that the preferential orientation can be changed from mono (0001) surface to a mixture of two components of (0001) and (10
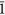
3) by adjusting the deposition parameters. It is well known that the (0001) plane has a relatively lower surface energy than the (10
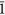
3) plane, and thus, the growth of Mg(0001) is much preferred^20^. However, the adsorption and occlusion of Ar gas strongly influence Mg atoms transfer. Thus, it is possible to tune the preferential growth and the structure of the magnesium film by adjusting the Ar pressure. The increase of Ar gas pressure could slow down the sputtered Mg atoms due to the collisions that occurred while moving to the substrate, which leads to relatively low energy bombardment on the surface of the growing thin film compared to high vacuum conditions. This may lower the diffusion ability of the atoms and favor the growth of high energy surface plane. Another possible reason is that the gas could be preferentially adsorbed on the high surface energy plane to reduce the surface energy, thus motivates the growth of high index planes. On another side, Lee^5^ reported that in the case when gas pressure is too high, the total-adsorption occurred by the increased gas particles. The nucleation of Mg is easily realized than the nucleus growth from the deposition. For this reason, the amount of (10
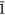
3) preferential orientation can be decreased. In this experiment, the 5.6 × 10^−1^ Pa pressure of Ar gas might be high enough to restrain the Mg(10
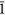
3) growth as shown in Fig. 1 (b). There should be an optimum conditions to fabricate highest ration of Mg(10
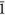
3), which will be a further study topic of future. From XRD patterns, it is also revealed that the width at half maximum of the Mg peaks is rather wide, which means the very small grain size of Mg, about 150 nm, as estimated by using the Scherer equation.

Transmission electron microscope (TEM) is used to study the microstructure of Mg films. Figure 1 (d) is the bright field image of the film, in which it shows the film is about 1900 nm thick. It is clearly shown that the Mg layer is composed of bundles. Nearly parallel columnar crystallites consisting of the bundles are nucleated and grow normally to the MmM_5_ substrate. Figure 1(e) is for a Mg layer with a (0001) preferential orientation; the long axis direction of the columnar crystal is parallel to the [0001] orientation of the Mg. Many stacking fault defects are observed in the Mg crystallites. Figure 1(f) is the bright field image taken from a Mg layer with coexisting (0001) and (10
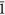
3) preferential orientations. The long axis direction of the columnar crystal with stacking faults is parallel to the [0001] orientation of Mg and the other part with no defects is parallel to the [10
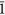
3] orientation. Interestingly, the density of stacking faults in the coexisting preferential orientation is lower than that in the pure (0001) preferential orientation thin film. This is an indication that the strain and stress in the thin film with coexisting (0001) and (10
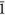
3) preferential orientations is smaller than that in the pure (0001) orientation thin film.

**Figure 2:**
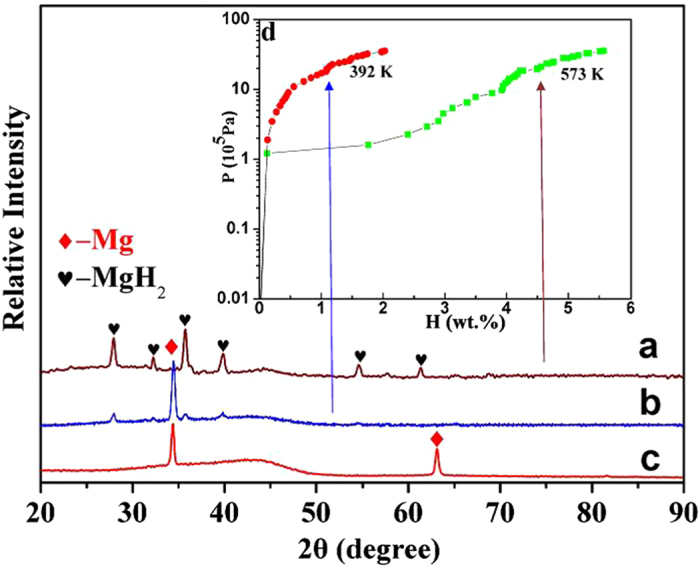
X-ray diffraction (XRD) patterns of Mg thin film hydrogenated at (**a**) 573 K, (**b**) 392 K and (**c**) as prepared, respectively. (**d**) PCI curves of Mg thin film measured at 573 K and 392 K.

To reveal the effect of crystallographic orientations on the hydrogen absorption of the thin films, hydrogenation is performed at different temperatures with coexisting Mg(0001) and (10
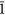
3) preferential orientations deposited using 250 W at 1.4 × 10^−1^ Pa Ar gas pressure. We should mention that the hydrogen absorption of the MmM_5_ buffer layer was neglected as it is very thin. Figure 2 (a) and (b) are the XRD patterns of the Mg thin films hydrogenated at 573 K and 392 K, respectively. The XRD pattern of the as-prepared film, Fig. 2(c), is also included for comparison. Figure 2 (b) shows the XRD patterns of the Mg thin film hydrogenated at 392 K. Compared with Fig. 2 (b) and (c), it is clearly stated that the original Mg diffraction peak for (10
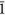
3), as shown in Fig. 2 (c), disappears and the MgH_2_ peaks appear while the (0001) peak still exists as shown in Fig. 2 (b). This result confirms that the columnar Mg with a (10
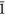
3) orientation has been hydrogenated at a temperature of 392 K, but the Mg layer with the (0001) orientation cannot be hydrogenated. Increasing the temperature to 573 K, the (0001)Mg diffraction peaks disappear, and only MgH_2_ peaks exist in the XRD patterns, as shown in Fig. 2(a), which means that the [0001] oriented columnar Mg completes the hydrogenation at this temperature. Figure 2(d) shows PCI curves of the Mg film measured at different temperatures. It is seen that the hydrogen absorption content is 2.3 wt.% at 392 K. This is because only Mg with a(10
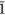
3) orientation absorbs the hydrogen under this condition. When the hydrogenation temperature increases to 573 K, the maximum hydrogen absorption content of the Mg film reaches 5.6 wt.%, which means that both crystallites with (10
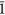
3) and (0001) orientations are hydrogenated. The above experimental results prove that the Mg with the (10
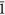
3) plane parallel to the surface can be hydrogenated much easier than that with the (0001) plane.

In order to clarify the mechanism of the penetration difference of H in the different orientations of thin films, the *ab initio* calculations were performed. Our calculations are performed using the Vienna *ab initio* Simulation Package^21^,^22^. The Perdew-Wang (PW91^23^) functional are used as the exchange-correlation potential with generalized-gradient approximation (GGA^24^) and projector-augmented wave (PAW^25^) potentials. Mg 3*s*^2^ and H 1*s*^1^ are treated as valence electrons in the PAW pseudo-potentials. The plane-wave cutoff energy is set to be 310 eV. Mg(10
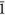
3) surface is modeled by a sixteen-atom-layer slab with a 15 Å vacuum. The slab lattice constants are *a* = 3.04 Å and *b* = 9.453 Å with an inclusion angle of 99.25º. A 2 × 1 unit cell is adopted for calculations of H adsorption energies, and an 1 × 1 one was for Nudged Elastic Band (NEB^26^) barriers. For the Brillouin-zone integration, a 6 × 4 × 1 Monkhorst-Pack *k*-point sampling is used for calculations of H adsorption energies (*E*_ad_), and a 6 × 2 × 1 one is for NEB barrier calculations^27^. The topmost twelve Mg layers are allowed to relax in both *E*_ad_ and NEB calculations. In *E*_ad_ calculations, the system are fully relaxed until the forces on the atoms are less than 0.02 eV/Å and the total energy change between two steps is less than 1 × 10^−4^ eV. In each NEB calculations, three images between the initial and final configurations are employed with a tolerance on forces of 0.05 eV/Å.

**Figure 3:**
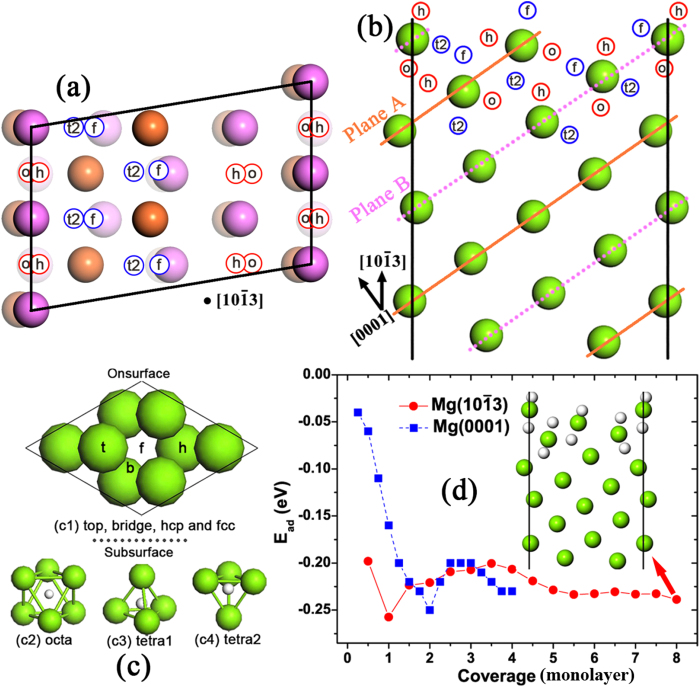
H adsorption configurations and energies on a (2 × 1) Mg (10
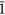
3) unit cell. (**a**) Vertical and (**b**)side views of Mg (10
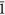
3) slab with possible H adsorption sites. For a guide to the eye, Mg atoms in part (**a**) are distinguishably colored with depth cue to correspond to A and B planes shown in part (**b**). In parts (**a**) and (**b**), the number of Mg atomic layers with H adsorption sites considered reaches up to 4.The adsorption sites on Mg (10
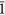
3) could be seen as those on its constituent Mg (0001) planes, which are the same as the real Mg (0001) slab, as shown in part (**c**), where seven possible adsorption sites are abbreviated to t, b, h, f, o, t1 and t2. For convenient discussion, the nomenclature of H adsorption sites on Mg (10
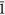
3) slab is allying this way: A^f^_1_or B^o^_2_, which means the first outmost fcc site on Plane A or the second outmost octa site on Plane B.(**d**) A comparison between adsorption energies on Mg (0001) and Mg (10
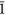
3)with different coverage, while the inset illustrates the relaxed configuration at coverage of 8 monolayer, where an adsorption configuration of A^f^_1_ + B^h^_1_ + B^h^_2_ + A^f^_2_ + B^o^_1_ + A^t2^_1_ + A^t2^_2_ + B^t2^_2_is adopted, on Mg(10
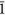
3). The adsorption energy *E*_ad_ is calculated by *E*_ad_ = [*E*_Mg(10_
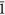
_3) + *x*H_ – (*E*_Mg(10_
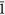
_3)_ + (*x*/2)*E*_H2_)]/*x*, where *x* is the number of adsorbed hydrogen atoms in the system, *E*_Mg(10_
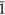
_3) + *x*H_, *E*_Mg(10_
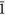
_3)_ and *E*_H2_ are the total energies of the optimized Mg-H adsorption system, the clean Mg (10
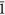
3) slab and gas-phase H_2_molecule. The Mg (0001) data is from a previous study^29^.

For hydrogen uptake, H adsorption energies on Mg(10
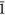
3) slab are considered. Firstly we focus on the H adsorption sites on Mg (10
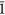
3) slab. According to the previous papers^28^,^29^, it was indicated that there were seven adsorption sites (four on-surface and three sub-surface sites) on a Mg(0001) layer, as shown in Fig. 3(c). Interestingly, the structure of Mg(10
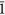
3) slab could be interpreted as consisting of groups of sliding Mg(0001) planes, which tend to form highly stable H-Mg-H trilayers^28^,^29^ during H uptake, with a *A*-*B*-*A*-*B*-… stacking pattern. Therefore, it is essential to check the H adsorption configurations similarities between Mg(10
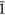
3) and Mg(0001) slabs. In practice, hydrogen atoms were gradually placed on both sides of outer most Mg[0001]-oriented Plane A or B, as shown in Fig. 3(a) and (b). The sites of the four outmost Mg atomic layers are fully considered. The adsorption energies with respect to hydrogen coverage *θ* (from 0.5 monolayer to 8 monolayer) on Mg(10
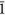
3) slab are calculated (shown in Fig. 3(d)) and compared with those^29^ on Mg(0001) slab.

We have considered a great deal of possible H adsorption configurations and sequences, and found that the atomic H adsorption configurations on Mg(10
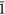
3) slab are analogous to those on Mg(0001) slab. Specifically, the on-surface fcc or hcp sites are the most energetically favored among seven possible on- and sub-surface adsorption sites on (0001)-oriented Plane A or Plane B leading to a relatively high penetration barrier for other atoms^30^. The adsorption energy is a difference between the final system energy (adsorbed atom or molecule plus the substrate) and the initial system energy (free atom or molecule plus the substrate). The more negative the adsorption energy is, the more stable the adsorbed system. For instance, the first H atom tended to take up the A^f^_1_ or B^h^_1_ sites, whose adsorption energies are −0.198 eV and −0.158 eV, respectively. As *θ* increases, H atoms would continuously occupy the other specific sites on (0001)-oriented Plane A or B It should be noted here that various adsorption sequences have to be considered as H coverage increases. After various configurations were considered and compared whose coverage ranged from 0.5 monolayer to 8 monolayer, there were two important observations: 1. The outermost sites were firstly occupied at initial H uptake; 2. Formations of H-Mg-H trilayers, although they are incomplete trilayers^29^, still persist on Mg(10
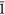
3) slab. Specially, the sequence of adsorption sites occupation from 0.5 monolayer to 8 monolayer tends to be in this manner: A^f^_1_ + B^h^_1_ + B^h^_2_ + A^f^_2_ + B^o^_1_ + A^t2^_1_ + A^t2^_2_ + B^t2^_2_. Namely, a clear trend of H-Mg-H trilayer formation could be observed, resulting in a facilitation in Mg-H system stabilizing. Moreover, as shown in Fig. 3(d), Mg(10
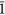
3) slab is more energetically favorable for H adsorption than Mg(0001) surface in the coverage ranges from 0.5 monolayer to 1.5 monolayer and from 2.5 monolayer to 3 monolayer. Especially when *θ*≦1 monolayer, the energy difference is over 100 meV per hydrogen atom. This energy advantage indicates that the Mg(10
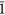
3) greatly favors initial hydrogen uptake. Precisely, this phenomenon can be attributed to the fact^31^ that the high-index Mg(10
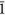
3) slab exhibits many dangling bonds including the basal and non-basal ones, which cause the slab instability. Thus, the saturation of Mg (10
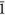
3) dangling bonds induced by the initial arrival of hydrogen atoms significantly increases the system stability and results in very negative adsorption energies. Moreover, the effect of saturation of Mg(10
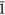
3) dangling bonds, especially the dangling basal bonds, is much more noticeable than that of the Mg (0001) slab, where no basal bond cutting is required upon slab creation^31^ Nevertheless, the *E*_ad_ differences between Mg (10
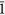
3)and Mg (0001) are narrowing as *θ* grows, and the energetic advantages are reversible when *θ* is between 1.5 monolayer and 2. monolayer or between 3.5 monolayer and 4 monolayer. It indicates that the formation of nearly or fully complete H-Mg-H trilayer^29^ on Mg(0001) slab is very energetically stable, which could not be surpassed by the incomplete H-Mg-H trilayers in Mg(10
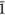
3) slab. Although the formation of complete H-Mg-H trilayers in Mg(0001) slab is more energetically advantageous at *θ* = 4 monolayer, the tiny *E*_ad_ difference between two slabs can be seen, i.e. merely 19 meV, thus the Mg(10
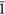
3) slab is still competitive. Considering the fact that the incomplete H-Mg-H trilayer on Mg(10
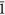
3) slab is far from being complete at *θ* = 4 monolayer, we continue the *E*_ad_ calculations on higher coverage, i.e. *θ* is greater than 4 monolayer. The results clearly show that the formation towards complete H-Mg-H trilayers further increases the system stabilities, manifested by the H adsorption energies of around − 0.225 eV in the coverage range from 4.5 monolayer to 8 monolayer. It means that Mg(10
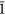
3) is very thermodynamically suitable for H adsorption. It should be noted that the optimized adsorption configuration on Plane B at *θ* = 8 monolayer is B^h^_1_ + B^h^_2_ + B^o^_1_ + B^t2^_2_, rather than the higher symmetric configuration of B^h^_1_ + B^h^_2_ + B^o^_1_ + B^o^_2_, where the Mg atom locates in the middle of B^h^_2_and B^o^_2_ H atoms and screens^28^ the electrostatic repulsion between B^h^_2_ and B^o^_2_H atoms leading to a system total energy lowering. It may be a hint that some phase transitions may happen to the system, which requires some future investigations to study. Besides, we should notice that the *E*_ad_ decreasing, as shown in the first two points of the red curve in Fig. 3 (d), is merely due to the subsequent H occupations of two equivalent most energetically favored A^f^_1_ sites, which strengthens^28^,^29^ the hybridizations between Mg *s* and H *s*, where the coverage is from 0.5 monolayer to 1 monolayer. Similar *E*_ad_ decreasing could be observed^28^ on the subsequent H occupation of four equivalent outermost fcc sites (one monolayer) on Mg(0001). No interaction between H atoms induces the variation of *E*_ad_ because the distance between H atoms is ~ 2 Å while the atomic and covalent radii of H atom are 0.79 Å and 0.32 Å. Overall, we find that Mg (10
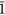
3) slab is very thermodynamically suitable for H uptake, either in the initial or continuing stage. After the thermodynamic advantages that Mg (10
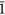
3) slab exhibits are revealed, one may be interesting in the H kinetics on Mg (10
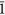
3) slab, which is also a major requisite for Mg (10
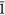
3) slab to be draw more sufficient attentions.

**Figure 4:**
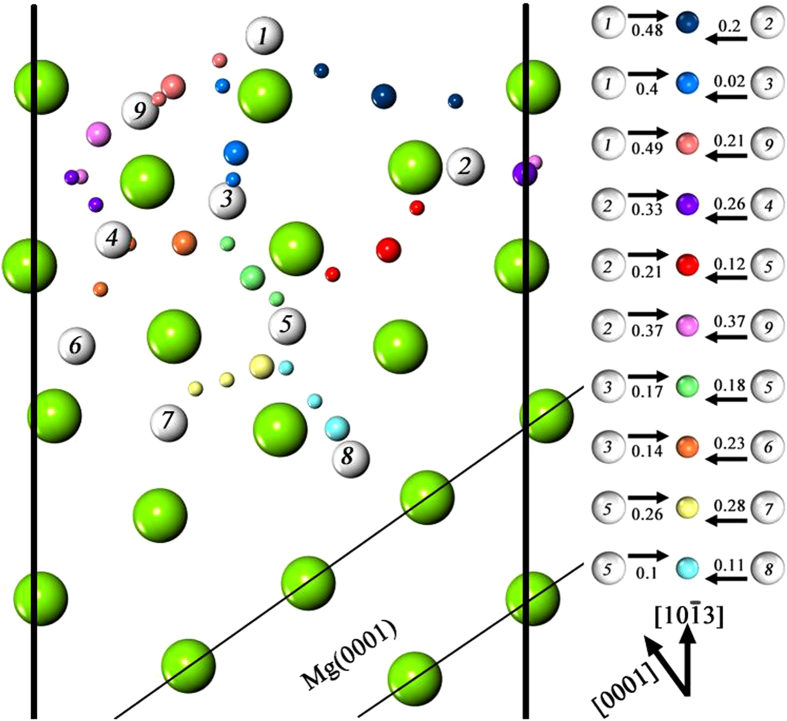
The pathways and energy barriers of hydrogen atomic penetration on an (1 × 1)Mg(103) slab from a side-view perspective. Here, the numbered H atoms occupy particular sites as described in Fig. 3(c) and (d). Various H migration pathways are in different colors with the white balls representing the initial or final state site, and Mg atoms are in green. Each NEB is calculated independently and the saddle points are shown slightly bigger in size. The energy barriers of both directions in each pathway are shown on the right. Of note, the energy barriers for H atomic penetration vertical to the first and second outermost Mg(0001) planes towards Mg bulk region on an (1 × 1) five-layer Mg(0001) slab are 0.78 eV (from A^f^ to B^f^) and 0.32 eV (from B^f^ to deeper A^f^), respectively. Meanwhile, the ones for H atomic migration parallel to the first and second outer most layers, 0.18 eV (from A^f^ to A^h^) are and 0.17 eV (from B^h^ to B^f^), respectively.

As shown in Fig. 4, hydrogen penetrations towards Mg bulk region on Mg (10
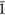
3) slab are different with those on Mg (0001) slab. Specifically, H migration energy barriers on Mg (10
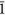
3) slab in surface region are slightly lower than those on Mg(0001) slab, and dramatically decrease as hydrogen atom penetrates more deeply towards Mg bulk region. Intriguingly, the pronounced decrease in the energy barrier can be attributed to the Mg (10
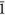
3) surface structure, which can be seen as consisting of groups of sliding Mg (0001) planes, as shown in Fig. 4. Recent investigation^31^ has confirmed the basal bonds in close-packed Mg(0001) plane have higher bonding energies than the non-basal ones due to the closer distance^32^ between the Mg atoms. Therefore, it requires more energy for hydrogen atoms to penetrate the Mg (0001) planes vertically. We performed the related energy barriers calculations for H migrations parallel to and H penetrations vertical to the first and second outermost Mg (0001) planes on an (1 × 1) five-layer Mg (0001) slab. The results show the energy barriers of the surface and first subsurface H migrations are 0.18 eV and 0.17 eV, respectively, while those of the H penetrations vertical to the first and second outermost Mg(0001) planes are 0.78 eV and 0.46 eV, respectively. The results indicate that the H migration parallel to Mg (0001) is much easier than the H penetration vertical to Mg (0001). However, the path across Mg (0001) on the Mg (0001) slab is the only feasible way for H to penetrate towards Mg bulk region. Therefore, this would possibly lead to not only high migration energies mentioned above but also unavoidable problems such as self-blocking^28^, i.e. the hydrogen migration passage may be blocked by the adsorbed H atoms and thus the H migration towards Mg bulk region is impeded. As a result, the sluggish H kinetics on Mg (0001) slab imminently happens.

Comparatively, Mg (10
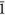
3) slab, with the utilization of non-basal passages between Mg (0001) layers is more advantageous for H penetration than Mg(0001) slab. As shown in Fig. 4, the space between sliding Mg (0001) planes on the Mg (10
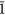
3) slab allows H penetration parallel to the Mg (0001) planes. Meanwhile, the weaker Mg bonding within Mg (0001) planes on Mg (10
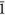
3) slab, which is reflected by its instability caused by more basal-bond cuttings, also facilitate the H penetration across the Mg (0001) planes compared with the Mg (0001) slab situation. Therefore, the lower hydrogen penetration energy barrier could be observed. It is found that the energy barriers for surface-region H penetration vertical to Mg (0001) planes are 0.48 eV from Site 1 to Site 2 and 0.4 eV from Site 1 to Site 3. These energy barriers are significantly lower than that of Mg (0001) slab, i.e. 0.78 eV, by 0.3 eV to 0.38 eV, respectively, though they are still relatively high partly due to the strong adsorption of hydrogen on the Mg (10
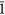
3) slab (see Fig.3). Besides the outmost layer penetration, the H penetrations on the Mg (10
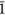
3) slab become much easier, as reflected by their low energy barriers (e.g. Site 3 to Site 6). Overall, the energy barriers are remarkably lower than the barriers of H vertical penetrations in Mg (0001) slab. Moreover, it could still be seen that the H penetration parallel to Mg (0001) plane is easier than the one vertical to Mg (0001). For instance, the penetration parallel to Mg (0001) from Site 2 to Site 5 had a barrier of 0.21 eV, while the one vertical to Mg (0001) from Site 2 to Site 4 had a barrier of 0.33 eV. Similar observations could be obtained by making comparisons between penetrations from Site 3 to Site 6 and from Site 3 to Site 5.The case of H penetration parallel to Mg (0001) from Site 5 to Site 7 is an exception when compared with the one from Site 5 to Site 8. In this case, it is found that the distance between two Mg atoms of adjacent Mg (0001) planes is so close, i.e. 3.07 Å, that more energy is required for hydrogen atoms to expel the Mg atoms to make the pathway. This should be an occasional situation. Mostly, the H penetration parallel to Mg(0001) plane is more thermodynamically favorable.

Therefore, we propose an effect of express penetration of H through the tilted close-packed-planes on Mg (10
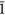
3) slab. The express penetration of H stems from the existence of the exclusive inter-close-packed-planar passages on Mg(10
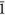
3), and the weak interaction and further distance between adjacent sliding Mg (0001) planes. Naturally, it could be utilized to facilitate the H penetration in Mg high-index slabs, rather than in the Mg low-index (0001) slab devoid of the exclusive inter-close-packed-planar passages. In the case of the Mg (10
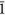
3) slab, the express penetration of H mainly contributes to the facilitation of H penetration towards Mg bulk region while the energy barrier is further lowered by the relaxations of Mg (10
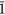
3) slab surface atoms, which expand the distances between Mg atoms and thus further improve the H penetration. In addition, the inter-close-packed-planar passages, to some extent, are capable of mitigating the self-blocking effect which causes sluggish H kinetics on Mg (0001) slab.

In summary, Mg materials with (0001) orientation and coexisting (0001) and (10
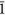
3) preferential orientations have been successfully synthesized by controlling the sputtering conditions. The hydrogen sorption property of Mg films is clearly correlated with its crystal orientations. The film with the Mg(10
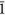
3) preferential crystal orientation parallel to the film surface is able to adsorb hydrogen at 392 K, while the film with the Mg(0001) preferential crystal orientation adsorbs hydrogen at 573 K. A maximum hydrogen absorption content of 5.6 wt.% is reached for the film. The theoretical calculations illustrate that the Mg (10
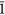
3) slab has low energy barriers for hydrogen penetration mainly due to the exclusive inter-close-packed passages in Mg (10
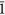
3) slab. Therefore, the Mg (10
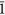
3) slab is capable of facilitating the formation of the precursor of the MgH_2_ layer in a hydrogen environment, which is supported by the experimental results.

## Methods

### Thin film fabrication

To confirm hypothesis that the kinetics of hydrogen sorption in Mg could be improved through its crystallographic features, it is necessary to prepare Mg films with a specific crystal surface. Mg thin films were deposited onto the MmNi_3.5_(CoAlMn)_1.5_ (MmNi_3.5_(CoAlMn)_1.5_ abbreviated as MmM_5_ here and after) buffer layer on a (0001) Si wafer by direct current magnetron sputtering using an Edwards Coating System (Model E306A). The Si substrate was pasted onto the substrate stage using a double stick tape and the substrate stage was cooled by circulating water. Two sputtering targets were used; one is a MmM_5_ alloy prepared by induction melting under the protection of pure Ar and the other is bulk Mg with a 99.99% purity. The base pressure in the working chamber was 5 × 10^−7^ mbar. In the deposition process, the MmM_5_ and Mg targets were alternatively positioned below the substrate and sputtered to deposit the MmM_5_ and Mg layers alternatively with designed thicknesses. Three-layered films were deposited with the starting and finishing layer being MmM_5_.

As is well known, it is fairly common for the (0001), (10
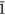
0), and (10
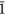
1) surfaces to preferentially grow, parallel to the substrate in the growth of hexagonal close-packed (hcp) metals via evaporation and sputtering^20^,^33^. No Mg thin film with (10
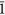
3), which is a high index plane, was fabricated under the normal conditions. In order to fabricate Mg (10
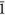
3), we tried different sputtering deposition parameters, such as various substrate materials including Si, sapphire, glass, and Ni, negative bias voltages, and various basepressures. The reason for these attempts is that the preferential orientation in the thin film can be controlled by particle bombardment and by the incorporation of one or several additional elements into the film^34^. For instance, the preferential orientation of films may shift from the (0002) to the (11
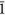
0) orientation as the negative bias voltage increases^35^. The substrate orientation change led to the growth orientation shift from (0002) to (10
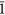
0) for sputter-deposited hexagonal close-packed (hcp) Ti^19^.

### Thin film characterization

X-ray diffraction (XRD) data of the samples were recorded by a Philips X'Pert MPD X-ray diffractometer with Cu Kα radiation. The transmission electron microscopy (TEM) was used to characterize the microstructures and orientations of Mg films (a Philips CM12 operated at 120 kV and a JEOL 3000 F equipped with an energy dispersive X-ray spectrometer (EDX) and operated at 300 kV). Hydrogen absorption was measured in a gas reaction controller (Advanced Materials Corporation) at different temperatures.

## Additional Information

**How to cite this article**: Ouyang, L. *et al.* Express penetration of hydrogen on Mg(10
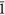
3) along the close-packed-planes. *Sci. Rep.* **5**, 10776; doi: 10.1038/srep10776 (2015).
